# High infiltration of CD209^+^ dendritic cells and CD163^+^ macrophages in the peritumor area of prostate cancer is predictive of late adverse outcomes

**DOI:** 10.3389/fimmu.2023.1205266

**Published:** 2023-06-26

**Authors:** Oscar Eduardo Molina, Hélène LaRue, David Simonyan, Hélène Hovington, Bernard Têtu, Vincent Fradet, Louis Lacombe, Paul Toren, Alain Bergeron, Yves Fradet

**Affiliations:** ^1^ Axe Oncologie, Centre de Recherche du CHU de Québec-Université Laval, Québec, QC, Canada; ^2^ Centre de Recherche sur le Cancer de l’Université Laval, Québec, QC, Canada; ^3^ Plateforme de Recherche Clinique et Évaluative, Centre de Recherche du CHU de Québec-Université Laval, Québec, QC, Canada; ^4^ Département de Chirurgie de l’Université Laval, Québec, QC, Canada

**Keywords:** prostate cancer, tumor immune microenvironment, tumor infiltrating immune cells, immunohistochemistry, prognostic biomarkers, dendritic cells, macrophages

## Abstract

**Introduction:**

Prostate cancer (PCa) shows considerable variation in clinical outcomes between individuals with similar diseases. The initial host-tumor interaction as assessed by detailed analysis of tumor infiltrating immune cells within the primary tumor may dictate tumor evolution and late clinical outcomes. In this study, we assessed the association between clinical outcomes and dendritic cell (DC) or macrophage (MΦ) tumor infiltration as well as with expression of genes related to their functions.

**Methods:**

Infiltration and localization of immature DC, mature DC, total MΦ and M2-type MΦ was analyzed by immunohistochemistry in 99 radical prostatectomy specimens from patients with 15.5 years median clinical follow-up using antibodies against CD209, CD83, CD68 and CD163, respectively. The density of positive cells for each marker in various tumor areas was determined. In addition, expression of immune genes associated with DC and MΦ was tested in a series of 50 radical prostatectomy specimens by Taqman Low-Density Array with similarly long follow-up. Gene expression was classified as low and high after unsupervised hierarchical clustering. Numbers and ratio of positive cells and levels of gene expression were correlated with endpoints such as biochemical recurrence (BCR), need for definitive androgen deprivation therapy (ADT) or lethal PCa using Cox regression analyses and/or Kaplan-Meier curves.

**Results:**

Positive immune cells were observed in tumor, tumor margin, and normal-like adjacent epithelium areas. CD209^+^ and CD163^+^ cells were more abundant at the tumor margin. Higher CD209^+^/CD83^+^ cell density ratio at the tumor margin was associated with higher risk of ADT and lethal PCa while higher density of CD163^+^ cells in the normal-like adjacent epithelium was associated with a higher risk of lethal PCa. A combination of 5 genes expressed at high levels correlated with a shorter survival without ADT and lethal PCa. Among these five genes, expression of *IL12A* and *CD163* was correlated to each other and was associated with shorter survival without BCR and ADT/lethal PCa, respectively.

**Conclusion:**

A higher level of infiltration of CD209^+^ immature DC and CD163^+^ M2-type MΦ in the peritumor area was associated with late adverse clinical outcomes.

## 1 Introduction

Prostate cancer (PCa) is the most frequently diagnosed cancer and the third leading cause of death in men from western countries (1, 2). Mortality by PCa after initial treatment with curative intent often occurs many years later, after a prolonged period of apparent cancer control. It is thus challenging to determine biomarkers that could predict such event. Even more difficult is to predict how the analysis of the primary cancer could lead to potential therapeutic interventions that could reduce the late mortality. Many hypotheses have been proposed to explain late recurrences, particularly at an older age. On the other hand, despite biochemical recurrences, many PCa patients survive until very old age without metastasis and die from other intercurrent diseases. The most common endpoint of biomarker studies in PCa is biochemical recurrence (BCR) but few studies have been able to relate biomarkers with long-term clinically important outcomes such as metastasis and PCa-related death.

The evaluation of the risk of recurrence and progression after initial treatment is currently based on clinicopathological factors such as tumor grade (Gleason score) and pathological stage (TNM), PSA level at diagnosis and presence of positive surgical margin (3, 4). However, there is variability in the individual risk of recurrence, progression and response to treatment in patients despite similar clinicopathological factors (5). There is growing evidence that an analysis of tumor infiltrating immune cells, and especially antigen presenting cells (APC) may help to better predict the evolution of cancers. APC are defined as a heterogeneous sub-group of immune cells that are responsible of the uptake, process, and presentation of antigens in order to activate naïve T-lymphocytes and trigger the adaptive immune response. Classical professional APC include dendritic cells (DC), macrophages (MΦ) and B cells. Acting at the interface between innate and acquired immune response, DC and MΦ play important roles in the antitumor immune response as evidenced by their association with clinical outcomes in various types of cancer (6–10).

In the tumor microenvironment, DC and MΦ can be found under different phenotypes corresponding to different functional activities. For example, immature DC, which express low levels of costimulatory molecules but express high levels of CD209 (DC-SIGN) have strong ability to detect and phagocyte the antigens (11). After phagocytosis, DC become mature and express high levels of costimulatory molecules such as CD83 (DC-LAMP) and migrate to lymph nodes where they will present the antigen to cognate T cells and stimulate their proliferation. Similarly, MΦ have been historically classified as pro-inflammatory M1 and alternatively anti-inflammatory M2 macrophages (12). However, because of their remarkable plasticity, tumor-associated MΦ can be modulated by the milieu and their interaction with tumor cells and thus they can be found in tumors as multiple subsets with mixed-phenotypes (13–15). This spectrum of phenotypes and functionality of tumor-associated MΦ complicates their analysis as there is a lack of specific markers for these states (16). CD68 is a pan-MΦ marker and the gold-standard marker to study human MΦ. Because of the increased scavenging capacities of M2 MΦ, the scavenger receptor CD163 is currently one of the most specific markers of M2 MΦ. In many cancers, CD163^+^ cells (M2-type MΦ) have been associated with poor prognosis (17–19). Analysis of these markers can provide valuable information on the contribution of MΦ to the anti-tumor response and clinical outcomes.

A few prior studies reported on the analysis of DC and MΦ infiltration in PCa and their association with clinical outcomes either using immunohistochemistry (IHC) (20–24) or using DC and MΦ-related gene expression analyses or inferential bioinformatic tools such as CIBERSORT (25–28). However, most if not all studies focus was on the tumor area or used patient cohorts with limited follow-up thus preventing assessment of the association of DC and MΦ with late, clinically important outcomes related to systemic progression of PCa.

In the present study we tested the hypothesis that the “initial host-tumor encounter” of APC with the PCa cells within the primary organ, could potentially influence late systemic progression and cancer mortality after radical prostatectomy. We investigated by IHC the presence of DC and MΦ with different phenotypes in the tumor core, tumor margin and normal-like immediate surrounding epithelial tissues of radical prostatectomy specimens from intermediate to high-risk PCa patients with very long follow-up. We also studied in a similar cohort the expression of genes associated with these APC and correlated them with the same clinical endpoints. Our study shows that the balance between immature and mature DC and the presence of high number of M2-type MΦ in the normal-like epithelium or tumor margin of PCa were independent predictors of lethal PCa in multivariate analyses adjusted for the known clinicopathological predictors. Moreover, the expression of a M2 MΦ-related gene and cytokine gene associated with APC was also predictive of lethal PCa.

## 2 Material and methods

### 2.1 Patients and tissues

This study was performed on radical prostatectomy specimens from 2 cohorts of men with localized PCa treated at CHU de Québec-Université Laval. The first cohort included 99 men who had surgery between March 1996 and November 1998. The radical prostatectomy specimens from this cohort were available as formalin-fixed and paraffin-embedded (FFPE) tissues and were used in IHC analyses. The second cohort was composed of 50 men who had radical prostatectomy between September 2004 and August 2009. Tissues from this cohort were available as frozen tissues embedded in OCT compound and were used in gene expression analysis using TaqMan^®^ Low Density Array (TLDA) technology. The database includes patients’ characteristics, pathological data and clinical follow-up allowing the assessment of time to BCR, to onset of definitive ADT, to development of castration-resistant prostate cancer (CRPC), to occurrence of metastases and to death from PCa. Time to lethal PCa was defined as either death from PCa and/or occurrence of metastasis and/or development of CRPC which inevitably leads to PCa mortality unless death occurs from intercurrent disease.

### 2.2 Immunohistochemistry

Five µm-thick sections of FFPE prostate tumors were prepared and dried overnight at 37°C. After deparaffinization, heat-induced antigen retrieval was performed in a PT Link (Dako, Burlington, ON, Canada) at 92°C for 20 min in either citrate buffer pH 6.1 for sections stained for CD209 and CD68 (Dako Code K8005: EnVision™ FLEX, Low pH) or Tris/EDTA, pH 9 for sections stained for CD83 and CD163 (Dako Code K8004: EnVision™ FLEX, High pH). Endogenous peroxidase activity was blocked by incubation in 3% peroxide solution for 10 min. The immunodetection was achieved using the IDetect super strain HRP polymer kit (ID labs, London, Ontario, Canada) as follows. Briefly, slides were incubated for 10 min at room temperature with Super block solution to block non-specific background staining. Then, primary antibody incubation was carried out at room temperature with monoclonal antibodies against CD83 (clone 1H4B, dilution 1:200, overnight incubation, Novocastra, Newcastle upon Tyne, England), CD209 (clone 120612, dilution 1:80, overnight incubation, R&D Systems, Minneapolis, MN), CD68 (clone KP1, dilution 1:800, 2 hr incubation, Abcam, Toronto, ON), and CD163 (clone 2G12, dilution 1:2000, 1 hr incubation, Abcam, Toronto, ON). After washes, slides were incubated for 30 min with HRP-Polymer Conjugate. Again, after washes, slides were incubated for 5 min with diaminobenzidine tetrahydrochloride solution to reveal bound antibodies and then rinsed, counterstained with hematoxylin, dehydrated and coverslipped using MM 24 low viscosity mounting medium (Leica Microsystems, Durham, USA). Each slide was digitalized using a Nanozoomer (Hamamatsu Photonics, Bidgewater NJ, USA) and visualized using the NDP.view2 software (Hamamatsu Photonics).

### 2.3 Immunohistochemistry scoring

For each slide, areas corresponding to tumor, tumor margin and normal-like adjacent epithelium were delineated. Then, in each of these areas, ten fields of view at 20x magnification (surface area of 0.460 mm^2^) were randomly selected for cell enumeration. The number of positive cells in each field of view was determined in a blinded manner either manually by two independent trained observers (OEM, HL) or semi-automatically using the Calopix software (TRIBVN Healthcare, Châtillon, France). In case of major disagreement on the scoring, the slides were re-evaluated until consensus was reached. For each marker, 10% of the slides were randomly selected and reviewed by an experienced pathologist (BT) to ensure accuracy of the data. Results were reported as number of positive cells per mm^2^.

### 2.4 Gene expression analysis

The tissue sections from the 50 frozen radical prostatectomy specimens used for the gene expression analysis were selected in order to have between 30 to 70% of tumor cellularity with presence of stroma and normal-like epithelium in the rest of the tissue. We also used normal prostate tissues from six cadaver organ donors that had no cancer after pathology review as control tissue for gene expression normalisation. For each eligible tumor or normal prostate, a group of ten 10 μm-thick sections were prepared for RNA extraction. H&E staining of sections made before and after the group of 10 sections was performed to confirm that selection criteria were maintained throughout the tissue depth. RNA extraction was performed on the ten 10 µm-thich sections using the Quick mirVana™ miRNA Isolation Kit from Ambion (ThermoFisher Scientific) in accordance with the manufacturer’s instructions. Following the extraction, RNA samples were treated for DNA contamination using an Ambion DNAfree™ kit (ThermoFisher) in accordance with the manufacturer’s instructions. Then, RNA was reverse-transcribed into first strand cDNA using the SuperScript^®^ VILO™ Invitrogen system (Life technologies, Waltham, MA, USA). TaqMan^®^ Array Micro Fluidic 384-Wells TLDA cards (Life technologies) were custom designed with pre-loaded gene-specific primer and probe sets for the analysis of the 14 selected immune gene targets related to APC (**Supplementary Table S1**) and two house-keeping genes *GUSB* and *PPIA* for mRNA normalization. Each cDNA sample (300 ng; 3 ng/μl) was added to an equal volume of 2X TaqMan Universal PCR Master Mix (Thermo Scientific) and 100 µL of the sample-specific PCR mix was added to the fill reservoir on the TLDA card. The card was centrifuged twice for one minute at 1200 rpm to distribute the sample-specific PCR reaction mix to the reaction wells. The card was sealed using the TaqMan Array Micro Fluidic Card Sealer (Thermo Scientific) and placed on microfluidic card thermal cycling block of a StepOnePlus™, 7900HT Fast Real-Time PCR Systems (Applied Biosystems). Thermal cycling conditions were as follows: 2 min at 50°C, 10 min at 94.5°C, 30 s at 97°C, 1 min at 59.7°C for 40 cycles. The target mRNA expression levels were normalized to *GUSB* and *PPIA* genes, the expression values of immune genes were calculated using ΔΔCT method, as recommended by the manufacturer. Each tumor gene expression value was then reported as a fold change of the same gene mean value in normal prostates. This resulting value was used for statistical analysis.

### 2.5 Statistical analyses

Baseline descriptive analyses were done using mean ± standard deviation (SD), frequency and percentage. The time-to-event variables were calculated from the date of radical prostatectomy to corresponding event date or to last follow-up date for censored patients. The number of positive cells per mm^2^ on IHC in each of the three compartments analyzed was grouped by quartiles for Kaplan-Meier analyses, with the log-rank test used for compared groups. Univariate and multivariable proportional hazards Cox regression models were fitted to estimate the crude hazard ratio (HR) and age, PSA, Gleason grade, T-stage, N-stage, and margin adjusted HR. Time to lethal PCa was defined as either death from PCa and/or occurrence of metastasis and/or development of CRPC. As analyses were exploratory, no adjustment for multiple testing was performed. The proportional hazards assumption of Cox models was checked using supremum test with 1000 replicates. The cause-specific proportional hazard Cox models were fitted to obtain stacked cumulative-risk function. For some specific analyses, bootstrap resampling procedure (≥1000 resamples) with unrestricted random sampling was used to investigate the internal validity of the Cox regression models. Pearson correlation was used to estimate relation between *CD163* and *IL12A* gene expression. Statistical analyses were performed using SAS Statistical Software v.9.4 (SAS Institute, Cary, NC, USA), with a two-sided significance level set at p < 0.05.

## 3 Results

### 3.1 Pathological characteristics and clinical outcomes of the two cohorts

**Figure 1:**
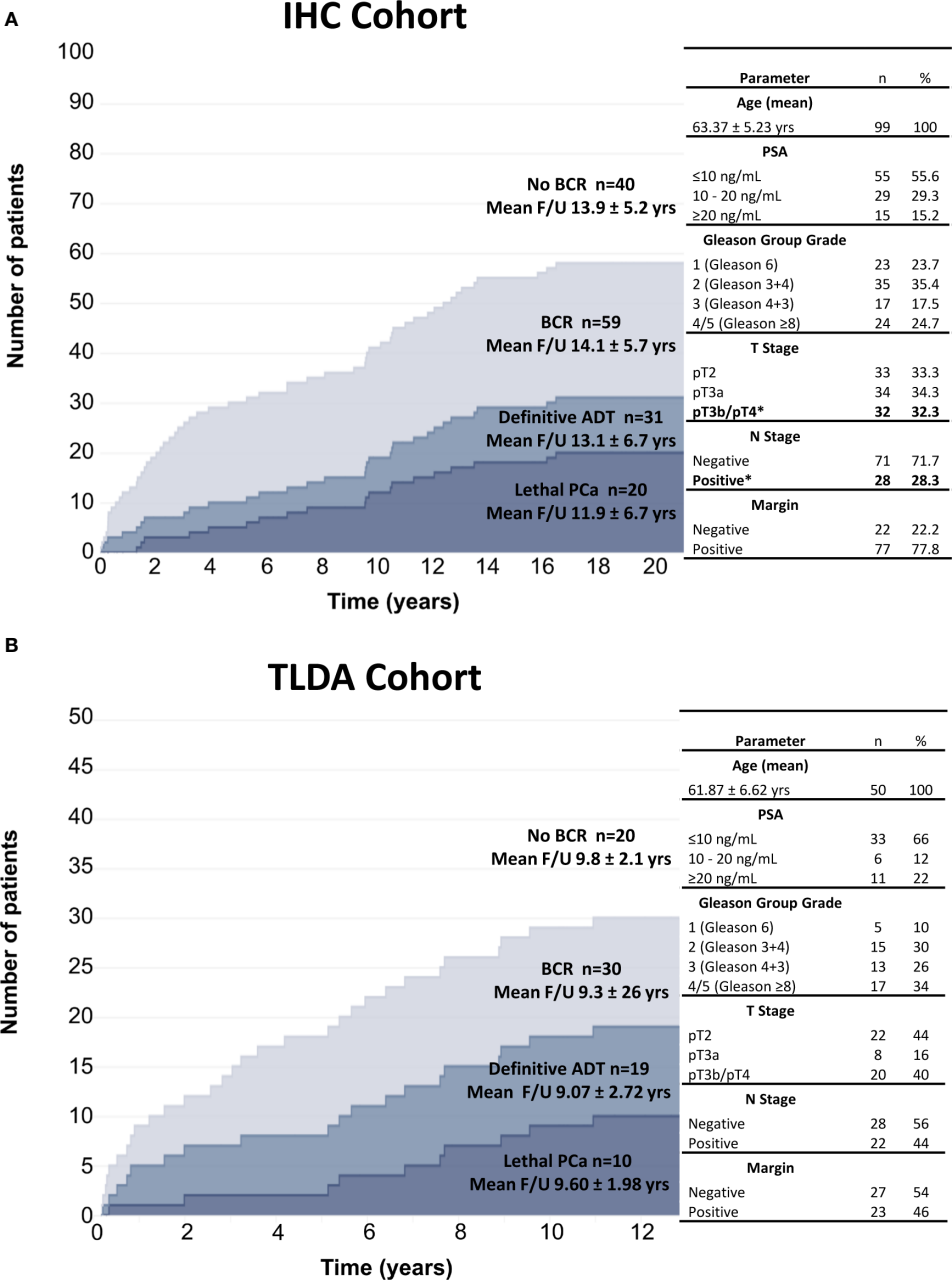
Graphic representation of the cohorts used for IHC analyses **(A)** and gene expression analyses by TLDA **(B)**. The number of patients and the mean follow-up time (yr) according to the clinical outcomes are indicated. Clinicopathologic factors significantly associated with definitive ADT and lethal PCa are identified by an asterisk.

The IHC cohort of FFPE radical prostatectomy specimens selected for analysis of DC and MΦ infiltration is characterized by a high proportion of patients with pathological features at high risk for recurrence. The long clinical follow-up (median of 15.5 years; mean of 14.0 years) provides for the assessment of late clinical outcomes such as the need for continuous definitive ADT and the occurrence of lethal PCa (**Figure 1A**).

Of the 99 patients, 59 experienced BCR at a median time of 6 years: 31 progressed to continuous ADT and 20 to lethal PCa. Of the other 28 BCR patients who did not progress to ADT or lethal PCa, 22 responded to salvage radiotherapy and 6 received short-term intermittent ADT. Multivariate Cox regression analyses were performed to assess the effect of the age, PSA levels, Gleason Group Grade categories, stage categories and presence of positive surgical margin on each of the clinical endpoints. Only pT3b/pT4 and lymph node metastasis were independent predictors of definitive ADT and lethal PCa (**Supplementary Table S2**). These parameters were included in all the multivariable analyses of IHC results.

The TLDA cohort of 50 patients with frozen prostatectomy specimens had high risk pathological characteristics similar to the IHC cohort (**Figure 1B**). Despite a shorter < 10 years mean follow-up, a similar proportion *i.e.* 30 out of 50 patients experienced BCR and 19 progressed to ADT of which 10 progressed to lethal PCa. Similarly, for the 11 BCR patients who did not progress to ADT or lethal PCa, 9 responded to salvage radiotherapy and 2 received short-term ADT. Given the small number of patients, only univariate analyses were performed on the TLDA results in relation to clinical outcomes.

### 3.2 Immunohistochemistry analyses: description of DC and MΦ infiltration

**Figure 2:**
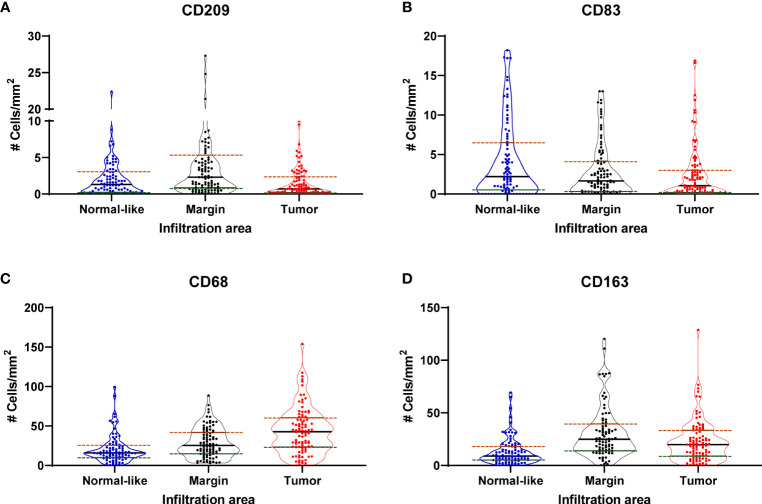
Violin plots showing the distribution of the density (cells/mm^2^) of CD209^+^ (immature DC) **(A)**, CD83^+^ (mature DC) **(B)**, CD68^+^ (total MΦ) **(C)** and CD163^+^ (M2-type MΦ) **(D)** cells in normal-like adjacent epithelium, tumor margin and tumor areas.

Immunohistochemical analyses were performed to identify CD209^+^ (immature DC), CD83^+^ (mature DC), CD68^+^ (total MΦ) and CD163^+^ (M2-type MΦ) cells in the 99 prostate tumors (**Figure 2**). The localization and characteristics of the positive cells in the different tissue compartments were described and the number of cells in tumor, tumor margin and normal-like adjacent epithelium areas was enumerated. The number of infiltrating cells was expressed as the mean number of cells by mm^2^. Overall, there was a greater number of MΦ than DC in all the compartments analyzed (**Figure 2**).

The CD209^+^ cells displayed a wide variation in staining intensities, but the overall staining level was low. These cells were rarely observed in the tumor and when present, it was usually more in the tumor stroma. In the normal compartment, CD209^+^ cells were also mostly in the stroma. The highest level of infiltration was at the invasive tumor margin, at the frontier adjacent to the stroma and normal areas. The infiltrating CD83^+^ cells were rather large, diffused and with apparent dendritic-like cellular extensions and had a high staining intensity. The tumor areas were scarcely infiltrated by these cells and the infiltration was also highest in the stroma of the tumor margin and the normal-like adjacent epithelium area.

The CD68^+^ and CD163^+^ cells were more abundant in the tumor than DCs and found both in the tumor epithelium and in the tumor stroma around the neoplastic glands. The tumor margins showed the highest infiltration, in particular with CD163^+^ cells which accumulated more at the frontier between the tumor and the stroma.

### 3.3 Association of IHC markers with clinical outcomes

**Figure 3:**
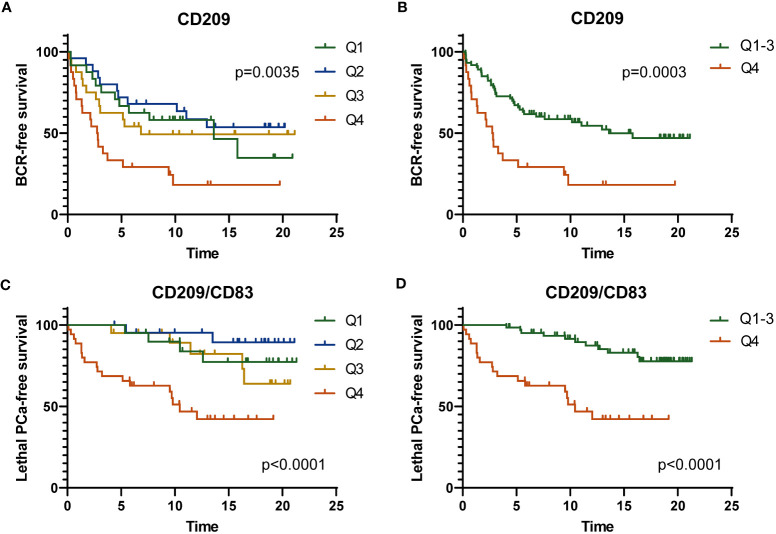
Kaplan-Meier curves showing the BCR-free survival according to the four levels of CD209^+^ cell infiltration (quartiles Q1 to Q4) **(A)** or according to low (Q1-Q3) and high level (Q4) of infiltration **(B)** in the tumor margin. Kaplan-Meier curves showing the lethal PCa-free survival according to the value of the CD209^+^/CD83^+^ ratio categorized as four levels of ratio (Q1 to Q4) **(C)** or according to low (Q1-Q3) and high ratio (Q4) of CD209^+^/CD83^+^ cell infiltration **(D)** in the tumor margin. The *p* value was estimated by the log-rank test.

The results of the number of positive cells per mm^2^ in each of the three compartments analyzed were stratified in quartiles (Q1 to Q4). Kaplan-Meier curves were generated for each marker in each compartment and for each of the three clinical outcomes. Since CD209^+^ immature DC will generate CD83^+^ mature DC upon antigen processing, we also analyzed the ratio of CD209^+^/CD83^+^ cell density for each patient and tissue compartment as a potential indicator of the balance between the two states. **Figure 3** shows two examples of Kaplan-Meier curves with each quartile and for highest (Q4) versus other lower quartiles (Q1-Q3) for CD209^+^ cell infiltration of the tumor margin and BCR outcome, and for the ratio CD209^+^/CD83^+^ cell infiltration of the tumor margin and lethal PCa. A high infiltration of CD209^+^ cells was associated with a lower BCR-free survival (**Figures 3A, B**) and a high ratio CD209^+^/CD83^+^ cells, i.e. more immature DC, was also significantly associated with lethal PCa (**Figures 3C, D**).

**Figure 4:**
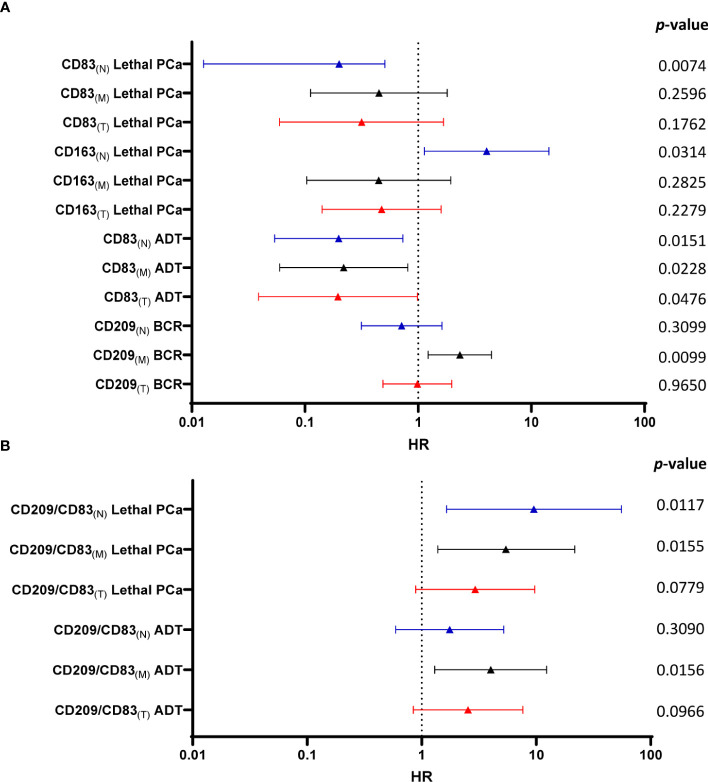
Forest plot of multivariate Cox regression analysis calculated HR, confidence intervals and *p*-values to predict the risk for each clinical outcome according to immune cells infiltrating the various tissue compartments **(A)**. The same analyses were performed with the value of CD209^+^/CD83^+^ cell density ratio in the various compartments **(B)**. Color code: blue - normal-like adjacent epithelium (N); black - tumor margin (M) and red - tumor (T) values.

Multivariable analyses were performed with adjustment for age, Gleason grade group, T stage, N stage and margin status for each marker in each compartment and for the clinical outcomes. Hazard ratio (HR) values and corresponding *p* values are listed in **Supplementary Table S3** and **S4**. **Figure 4A** shows in forest plots the various hazard ratio (HR) values, confidence intervals and corresponding *p* values for the significant markers predictive of clinical outcomes. As shown in the **Figure 4A**, a high vs low infiltration of CD209^+^ cells in the tumor margin was associated with a higher risk of BCR (HR=2.34, p=0.0099) while a high infiltration of CD163^+^ cells in the normal-like adjacent epithelium area was associated with a higher risk of lethal PCa (HR=4.03, p=0.0314). On the other hand, high density of CD83^+^ cells wherever in the tumor microenvironment was associated with a lower risk of the need for definitive ADT and high infiltration in the normal-like epithelium was associated with lethal PCa.

As the number of immature DC is potentially more significant when related to the number of mature DC, the same Cox regression analyses were performed with the CD209^+^/CD83^+^ cell density ratios. **Figure 4B** shows that a high CD209^+^/CD83^+^ cell density ratio in the tumor margin was associated with a higher risk for the need for definitive ADT (HR=4.02, p=0.0156) and with lethal PCa when the cells were in tumor margin (HR=5.43, p=0.0155) or the normal-like epithelium areas (HR=9.52, p=0.0117). The latter high HR was further validated by a bootstrap resampling procedure (HR=9.52; Interquartile HR 5.93-61.57)

### 3.4 Gene expression analyses

**Figure 5:**
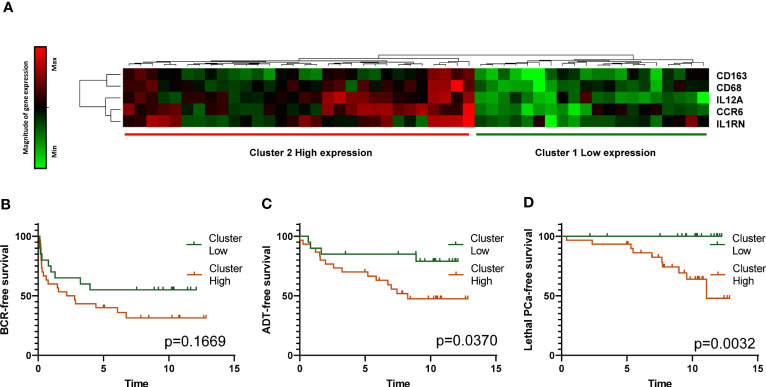
Analysis of APC-related immune gene expression by TLDA. Heatmap resulting from the unsupervised hierarchical clustering of the expression of five APC-related immune genes into two clusters corresponding to high and low expression of the genes is presented **(A)**. Gene expression determined by real time qRT-PCR with Taqman probes is shown in the heatmap. Each horizontal row represents the same gene product and each vertical row, each patient. Magnitude of expression from high (red) to low (green) is indicated by the colored bar. Kaplan-Meier survival curves showing the association of high and low expression of the five genes with survival without BCR, definitive ADT and lethal PCa are presented in panels **(B, C)** and **(D)**, respectively. The *p* values were estimated by the log-rank test.

The value of each of the 14 normalized immune gene mRNA levels for each tumor was analyzed as a ratio of expression relative to the average expression of the same gene in the six normal prostates using the ΔΔCT method. Unsupervised hierarchical clustering was performed by Pearson distance correlation followed by a complete-linkage algorithm to define molecular subgroups. Delta cycle threshold (Ct) values were used for this analysis and a heatmap was generated. A subgroup of five genes (*CD163, CD68, IL12A, CCR6* and *IL1RN*) provided the best separation of the patients in two groups and was used to generate a heatmap with low and high expression of the genes (**Figure 5A**). Kaplan-Meier curves were generated to assess the association of the expression of these genes with clinical outcomes. A high expression of the five genes was associated with a shorter time to the need for definitive ADT (*p*=0.0370) (**Figure 5C**) and to lethal PCa (*p*=0.0032) (**Figure 5D**) but not to BCR (**Figure 5B**).

**Figure 6:**
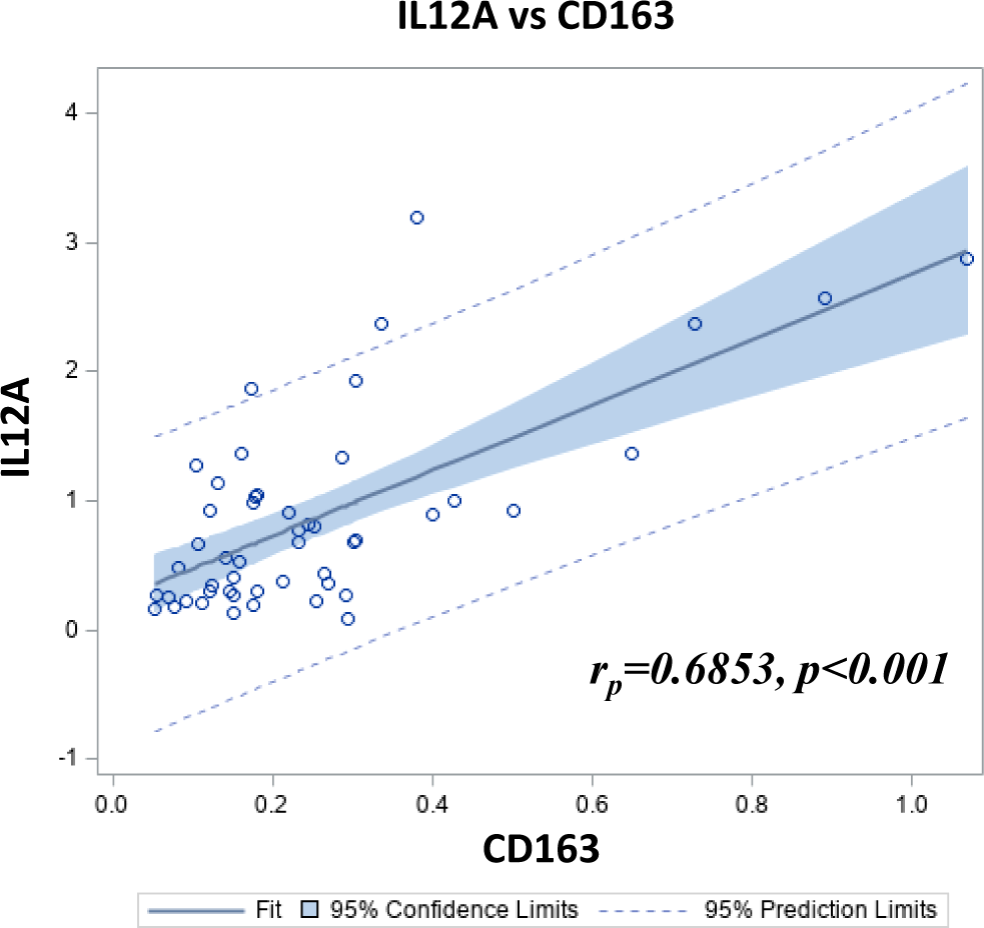
Scatterplot showing the Pearson correlation between *CD163* and *IL12A* relative gene expression in 50 PCa samples as determined by TLDA.

**Figure 7:**
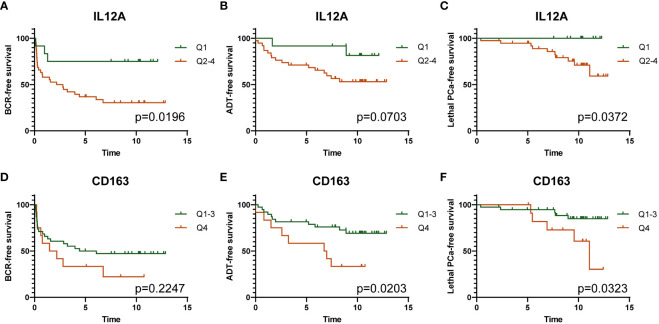
Kaplan-Meier survival analyses showing the association of low (Q1) and high (Q2-Q4) expression of *IL12A* gene with survival without BCR **(A)**, definitive ADT **(B)**, and lethal PCa **(C)**; and the association of low (Q1-Q3) and high (Q4) expression of *CD163* gene with survival without BCR **(D)**, definitive ADT **(E)**, and lethal PCa **(F)**. The *p* values were estimated by the log-rank test.

Among these genes, expression of *CD163* gene was correlated with that of *IL12A* (**Figure 6**). To assess the association of these genes with the progression of PCa, Kaplan-Meier curves were generated with data for these two genes individually, categorized as quartiles. **Figure 7** shows that a low expression of *IL12A* was associated with a longer BCR-free survival (*p*=0.0196) while a high expression of *CD163* was associated with a shorter time to definitive ADT *(p*=0.0203) and to lethal PCa (*p*=0.0323). These associations were confirmed by univariate Cox regression analysis. HR for these same associations were 0.26 (*p*=0.0298), 2.825 (*p*=0.0263) and 3.56 (*p*=0.0450), respectively (not shown).

## 4 Discussion

One clinical challenge in the management of PCa is to be able to identify those cancers that will progress to become lethal after initial treatment with curative intent. Clinicopathological factors are still the best to predict the evolution of PCa but they are limited since tumors with same characteristics can evolve differently. Hence, new biomarkers are needed to better prognosticate PCa.

Recurrence of PCa can be seen even ten years after radical prostatectomy as observed in our IHC cohort of patients with a median of 15.5 years of follow-up in whom 32% of BCR occurred after 10 years. This suggests that the growth of tumor cells that have escaped the prostate before the surgery is controlled to prevent immediate outgrowth. Some hypotheses have been proposed to explain these late recurrences, particularly at an older age. These include among others immune-senescence and cancer dormancy (29, 30). The concept of cancer dormancy has been coined to explain this phenomenon observed in some cancers, especially in prostate and breast cancers (30). The concept of tumor mass dormancy implies that there is an equal balance between increases in the number of cancer cells by proliferation and decreases as a result of cell death. Angiogenesis and immune-related mechanisms are believed to be the main factors contributing to the tumor mass dormancy (30).

It has been known for years that the immune system has the potential to recognize and kill cancer cells. However, tumors have developed several mechanisms to escape the immunosurveillance, including immunoselection and immune-subversion (31, 32). This results in various types of tumors with different immune cell infiltration profiles (33). The tumor cell-mediated immune-subversion implies that the normal functions of the immune cells are downplayed in favor of new functions that help promote tumor cell survival and growth. The immune contexture which is determined by the localization, density, composition, and functional state of the immune cells infiltrating the tumor can yield a great deal of information relevant to prognosis and prediction of therapeutic response (34).

Galon and colleagues were first to report (35–38) in colon cancer that the analysis of the interplay between the immune system and the primary cancer was more predictive of the short and medium term clinical outcome after surgery than the standard clinicopathological parameters. Other studies in breast cancer have also shown that the analysis of the immune infiltrating cells in the tumor may have prognostic implications (6, 39–41).

In PCa, there was a paucity of information about the prognostic value of the infiltration of tumors by DC and MΦ. However, Lie et al. reported a study on 151 PCa patients treated with radical prostatectomy with a median follow-up of 9 years which suggested that low infiltration by immature DC as detected by CD1a expression had a tendency (*p*=0.123) toward impaired BCR-free survival. There was no statistical significance in univariate and multivariate Cox regression analysis between the infiltration of CD1a^+^ immature DC and clinical outcome (biochemical failure, clinical failure, PCa-specific mortality and overall survival). It is interesting to notice that in this study the infiltration by CD1a^+^ immature DC in PCa samples was assessed by counting the number of positive cells in the tumor samples without taking into account the localization of the immune cells (42). We also analyzed the expression of CD1a in our IHC cohort but found very few cells being positive thus precluding any distinctive profile between individual patients (data not shown).

Lanciotti et al. reported in a cohort of 93 patients treated by radical prostatectomy a higher infiltration by CD68^+^ cells in tumor areas in organ confined PCa but a higher infiltration by CD163^+^ cells for patients with PCa with tumor extension. Although statistical analyses for Cox multivariate analyses did not find any statistical correlation between clinical outcome and CD68^+^ or CD163^+^ cell infiltration, there was a trend toward increased BCR failure rates for high infiltration of CD163^+^ cells (43). Also, Lundholm et al. analyzed a TMA of 234 PCa treated by TURP and found no statistical association for M1 MΦ infiltration and cancer specific survival. But a high infiltration by CD163^+^ cells was associated with a lower cancer-specific survival (*p*=0.002) when compared to a low infiltration. However, multivariate analyses adjusted for clinicopathological factors did not find any statistical correlation (44). In contrast, our results may have reached statistical significance given our selection of higher risk patients with more clinical events such as metastases and death related to PCa expected to occur. Meng et al. performed a bioinformatic study using the TCGA database comprising the gene expression profiles of 499 PCa samples and 52 controls to identify the profile of 22 types of immune cells using CIBERSORT. They reported an association with a shorter BCR-free survival for high density M2 MΦ compared to the low density (*p*=0.025) in log rank analyses. No statistical correlation for M0 and M1 MΦ were found (27). In 2020, Yuri et al. presented a study of tissue samples from 54 patients diagnosed with PCa treated by either radical prostatectomy or transurethral resection of the prostate. Six hot spots in peritumoral tissue per patient were considered for the analysis *i.e.* areas most positive for CD68 examined at ×400 magnification to obtain the average value of the 6 areas around the cancer foci. High CD68^+^ cell infiltration was associated with shorter duration of survival in univariate (HR=4.47; 95% CI:1.97–10.15, *p* =<0.001) and multivariate (HR =3.51; 95%CI: 1.49–8.26, *p* =<0.004) Cox regression analyses (45). More recently, Andersen et al. also showed that high CD68^+^CD163^+^CD20^-^ cell infiltration in the epithelium area was significantly associated with BCR in multivariate Cox regression analyses in two different cohorts (46). The authors also validated by deconvolution analysis of RNAseq data from 99 bulk PCa tissue samples the association between MΦ and BCR.

Since immunohistochemistry analyses is limited to a few immune markers available for this approach, we have performed a gene expression analysis to include genes of cytokines, chemokines and transcription factors that could also be predictive of the clinical outcomes. The TLDA analysis showed that a combination of 5 genes expressed at high levels correlated with a shorter survival without ADT and lethal PCa. The presence of *CD163* among these five genes correlated with adverse outcomes, supports the association between CD163^+^ M2-type MΦ infiltration and higher risk of adverse PCa outcomes observed within the immunohistochemistry cohort. Interestingly, *CD163* gene expression was correlated with that of *IL12A*. This result, suggesting that a high *IL12A* gene expression was associated with a poor prognosis, was *a priori* surprising since IL-12 is a cytokine that is known to have immunostimulating functions and has been shown to have a great potential for cancer immunotherapy (47). However, the *IL12A* gene does not code for the entire IL-12 cytokine but only for its subunit alpha, also known as p35. The subunit alpha binds to the subunit beta, also known as p40, to create the IL-12 cytokine. However the subunit alpha can also bind to the Epstein-barr virus-induced gene 3 (Ebi3) protein to form the IL-35, a cytokine known to promote tumor growth and metastasis by converting resting B and T cells into IL-10- and IL-35-producing regulatory B (Breg) and T (Treg) cells (48). IL-35 has been recently shown to be associated with the tumorigenesis and promotion of PCa (49, 50). The association of *IL12A* gene expression with poor prognosis might therefore be related to IL-35 in PCa. Further studies are required to assess the relationship between *IL12A* and *CD163* gene expression and IL-35 in PCa.

In the present study we sought to determine whether there was an association between the infiltration of DC and MΦ and clinical outcomes, taking into account the localization within the tumor, the margin and surrounding normal tissue. We used for each cell type, markers that allowed us to distinguish cells with specific phenotypes and functionality (*e.g.* mature vs immature DC). Positive immune cells were observed in all areas but in different proportion for certain types of cells as for example CD209^+^ immature DC and CD163^+^ M2-type MΦ were more abundant at the tumor margin. Higher CD209^+^/CD83^+^ cell density ratio in the margin was associated with higher risk of the need for definitive ADT and lethal PCa while higher levels of CD163^+^ cells in the normal-like adjacent epithelium was associated with a higher risk of lethal PCa. Following these observations, one important question to answer is how these cells contribute to the tumor development and progression. T cells are the main effector of the antitumor response and their anti-tumor functions are directly regulated by APC through either direct contact or through the effect of soluble secreted factors or extracellular vesicles (51). For example, tumor-associated MΦ release the immunosuppressive cytokine IL-10 or induce local depletion of arginine and tryptophan which are needed for T-cell activation and polarization (52, 53). They also secrete TGF-ß which promote PD-1 expression on CD4^+^ T cells and induce the conversion of CD4^+^ T cells into Tregs and their infiltration in tumors (54). In PCa, high levels of Tregs in tumors were associated with aggressiveness and poor prognosis (46, 55). These associations suggest that targeting Tregs and tumor-associated MΦ might be an effective anti-tumor approach. Etzerodt et al. showed in an experimental model of melanoma resistant to PD-1 blockade that depletion of CD163^+^ macrophages resulted in a massive infiltration of activated T cells and ultimately, in tumor regression (56). MΦ-targeting immunotherapy approaches consisting in reprogramming of tumor-associated MΦ are being developed and hopefully will lead to more effective tumor treatment strategies (57).

One strength of the present study is the maturity of follow-up in the two cohorts allowing to correlate immune infiltrations with systemic progression. As shown in our cohorts, BCR as an endpoint does not always translate into late progression, as up to one third of these patients could be rescued by salvage or adjuvant radiotherapy or short-term ADT. Also, the ratio of immature to mature DCs which allows to quantify the balance between the two states was shown to have additional prognostic potential. Despite the relatively small number of patients, the maturity of the clinical follow-up and our detailed analysis provided very strong indicators of late events in adjusted analyses taking into account the clinicopathological predictors. This type of analysis could be performed on prostate biopsies before surgery to identify a “missed encounter” between the host immune system and the PCa. These patients could potentially benefit from intra-prostatic and systemic neoadjuvant interventions meant to revert the suppressed tumor micro-environment, results that could be assessed on the prostatectomy specimens as a first short-term endpoint.

## 5 Conclusion

A higher level of infiltration of CD209^+^ immature DC and CD163^+^ M2-type MΦ in the peritumor area was associated with a higher risk of late clinical outcomes such as the need for definitive ADT and lethal PCa. These results suggest that more investigations of the prognostic value of CD209^+^ and CD163^+^ cells in the peritumor areas should be performed in larger cohorts to confirm the results and evaluate their utility to identify patients at risk for clinically important outcomes.

## Data availability statement

The raw data supporting the conclusions of this article will be made available by the authors, without undue reservation.

## Ethics statement

This study involving human participants was reviewed and approved by the Comité d’éthique à la recherche du CHU de Québec-Université Laval (Project 2012-1059). The patients/participants provided their written informed consent to participate to the local urological biobank (URO-1 Biobank) allowing the use of their tissues and clinico-pathological data for this study.

## Author contributions

Conceptualization, OM, HL, LL and YF. Methodology, OM, HL, LL and YF. Experimentation, data acquisition and initial analyses, OM, HL, HH, and BT. Statistical analyses, OM, DS and YF. Resources, HL, HH, AB and YF. Writing—original draft preparation, OM, AB and YF. Writing—review and editing, OM, HL, DS, HH, PT, BT, VF, LL, AB and YF. Supervision, HL, AB and YF. Funding acquisition, HL, LL, BT, VF, AB, YF All authors have read and agreed to the published version of the manuscript.
