# Supplementary material for: High infiltration of CD209+ dendritic cells and CD163+ macrophages in the peritumor area of prostate cancer is predictive of late adverse outcomes
Source: Front Immunol. 2023 Jun 26;14:1205266. doi: 10.3389/fimmu.2023.1205266 (PMC10331466; doi:10.3389/fimmu.2023.1205266)
Supplement: Supplementary file 1 [file DataSheet_1.pdf]

Supplementary Material

1 Supplementary Tables

Supplementary Table S1. Selected genes for TLDA analyses.

Gene ID	Gene name	Association
CCL1-Hs00171072_m1	Chemokine (C-C motif) ligand 1	Mature DC
CD83-Hs00188486_m1	CD83 molecule	Mature DC
CCR6-Hs01890706_s1	Chemokine (C-C motif) receptor 6	Immature DC
CD1A-Hs00381754_g1	CD1a molecule	Immature DC
CD1E-Hs00951508_g1	CD1e molecule	Immature DC
CD209-Hs01588349_m1	CD209 molecule	Immature DC
CD68-Hs02836816_g1	CD68 molecule	MΦ
GDF15-Hs00171132_m1	Growth differentiation factor 15 (MIC-1)	MΦ
IDO2-Hs01589373_m1	Indoleamine 2,3-dioxygenase 2	M1 MΦ
IL12A-Hs01073447_m1	Interleukin 12A	M1 MΦ
CD163-Hs00174705_m1	CD163 molecule	M2 MΦ
IL1RN-Hs00893626_m1	Interleukin 1 receptor antagonist	M2 MΦ
IL8-Hs00174103_m1	Interleukin 8	Inflammation
PTGS2-Hs00153133_m1	Prostaglandin-endoperoxide synthase 2 (Cox-2)	Inflammation

Supplementary Table S2. Baseline characteristics of the IHC cohort and multivariate Cox regression analyses according to the three clinical outcomes.

			BCR		Definitive ADT		Lethal PCa	
**Parameter**	**n**	**%**	**HR (95% CI)**	***p*-value**	**HR (95% CI)**	***p*-value**	**HR (95% CI)**	***p*-value**
**Age (mean ± SD)**								
63.37 ± 5.23 yrs	99	100.00	0.98 (0.92-1.04)	0.4105	0.96 (0.89-1.04)	0.2980	1.01 (0.93-1.10)	0.8768
**PSA**								
≤10 ng/mL ref*	55	55.56	1		1		1	
10 - 20 ng/mL	29	29.29	1.37 (0.67-2.79)	0.3936	2.13 (0.74-6.15)	0.1619	0.65 (0.17-2.45)	0.5237
≥20 ng/mL	15	15.15	1.49 (0.65-3.40)	0.3500	1.40 (0.44-4.47)	0.5671	1.02 (0.29-3.64)	0.9710
**Gleason Group Grade**								
1 (Gleason 6)	23	23.23	0.64 (0.25-1.65)	0.3577	-	-	-	-
2 (Gleason 3+4) ref*	33	33.33	1		1		1	
3 (Gleason 4+3)	17	17.17	1.40 (0.61-3.19)	0.4279	0.79 (0.23-2.66)	0.7033	2.83 (0.65-12.35)	0.1663
4/5 (Gleason ≥8)	24	24.24	1.27 (0.58-2.76)	0.5523	1.57 (0.55-4.46)	0.3978	2.24 (0.67-7.53)	0.1931
**T Stage**								
pT2	33	33.33	0.53 (0.23-1.21)	0.1323	2.41 (0.57-10.23)	0.2332	0.80 (0.07-9.14)	0.8580
pT3a ref*	34	34.34	1		1		1	
pT3b/pT4	32	32.32	1.73 (0.83-3.61)	0.1458	**4.18 (1.16-15.10)**	**0.0290**	**6.40 (1.35-30.41)**	**0.0196**
**N Stage**								
Negative ref*	71	71.72	1		1		1	
Positive	28	28.28	0.85 (0.43-1.70)	0.6474	**4.28 (1.61-11.37)**	**0.0036**	**3.30 (1.07-10.16)**	**0.0379**
**Margin**								
Negative ref*	22	22.22	1		1		1	
Positive	77	55.56	1.52 (0.72-3.21)	0.2713	0.73 (0.26-2.04)	0.5457	5.16 (0.96-27.58)	0.0552

* Reference category

Supplementary Table S3. Multivariate Cox regression analyses calculated HR to predict the risk for each clinical outcome according to a high (Q4) vs low (Q1-Q3) density of cells infiltrating the normal-like adjacent epithelium, tumor margin and tumor areas.

Clinical outcome	Marker	Localisation	HR (95% CI)	*p*-value
**BCR**	**CD209**	**Normal**	**2.34 (1.23-4.44)**	**0.0099**
	CD209	Margin	1.43 (0.72-2.85)	0.3099
	CD209	Tumor	0.98 (0.49-1.98)	0.9650
	CD83	Normal	0.56 (0.27-1.18)	0.1262
	CD83	Margin	0.86 (0.43-1.72)	0.6736
	CD83	Tumor	1.04 (0.51-2.09)	0.9176
	CD163	Normal	1.04 (0.48-2.23)	0.9266
	CD163	Margin	0.88 (0.40-1.93)	0.7458
	CD163	Tumor	1.16 (0.61-2.22)	0.6528
	CD68	Normal	0.85 (0.40-1.81)	0.6783
	CD68	Margin	0.80 (0.39-1.63)	0.5449
	CD68	Tumor	0.93 (0.45-1.93)	0.8395
**Definitive ADT**	CD209	Normal	0.30 (0.08-1.09)	0.0672
	CD209	Margin	0.94 (0.32-2.74)	0.9091
	CD209	Tumor	1.18 (0.37-3.76)	0.7805
	**CD83**	**Normal**	**0.20 (0.05-0.73)**	**0.0151**
	**CD83**	**Margin**	**0.22 (0.06-0.81)**	**0.0228**
	**CD83**	**Tumor**	**0.20 (0.04-0.98)**	**0.0476**
	CD163	Normal	2.05 (0.75-5.59)	0.1598
	CD163	Margin	1.62 (0.57-4.59)	0.3663
	**CD163**	**Tumor**	**2.56 (1.00-6.54)**	**0.0500**
	CD68	Normal	2.46 (0.85-7.09)	0.0954
	CD68	Margin	2.27 (0.89-5.81)	0.0866
	CD68	Tumor	0.44 (0.15-1.29)	0.1328
**Lethal PCa**	CD209	Normal	1.16 (0.29-4.57)	0.8334
	CD209	Margin	0.90 (0.29-2.79)	0.8598
	CD209	Tumor	1.43 (0.38-5.38)	0.5940
	**CD83**	**Normal**	**0.08 (0.01-0.51)**	**0.0074**
	CD83	Margin	0.45 (0.11-1.81)	0.2596
	CD83	Tumor	0.32 (0.06-1.68)	0.1762
	**CD163**	**Normal**	**4.03 (1.13-14.29)**	**0.0314**
	CD163	Margin	0.45 (0.10-1.94)	0.2825
	CD163	Tumor	0.47 (0.14-1.59)	0.2279
	CD68	Normal	2.34 (0.76-7.19)	0.1368
	CD68	Margin	1.67 (0.63-4.44)	0.3064
	CD68	Tumor	1.00 (0.29-3.47)	0.9993

Supplementary Table S4. Multivariate Cox regression analyses calculated HR to predict the risk for each clinical outcomes according to a high (Q4) vs low (Q1-Q3) ratio of $CD209^{+}/CD83^{+}$ cells infiltrating the normal-like adjacent epithelium, tumor margin and tumor areas.

.

**Clinical outcome**	**Marker**	**Localisation**	**HR (95% CI)**	***p*-value**
**BCR**	CD209/CD83	Normal	1.36 (0.64-2.91)	0.4218
	CD209/CD83	Margin	1.87 (0.93-3.76)	0.0806
	CD209/CD83	Tumor	1.10 (0.56-2.17)	0.7715
**Definitive ADT**	CD209/CD83	Normal	1.76 (0.59-5.21)	0.3090
	CD209/CD83	**Margin**	**4.02 (1.3-12.35)**	**0.0156**
	CD209/CD83	Tumor	2.54 (0.85-7.63)	0.0966
**Lethal PCa**	CD209/CD83	**Normal**	**9.52 (1.65-55.56)**	**0.0117**
	CD209/CD83	**Margin**	**5.43 (1.38-21.74)**	**0.0155**
	CD209/CD83	Tumor	2.93 (0.89-9.71)	0.0779

2 Supplementary Figures

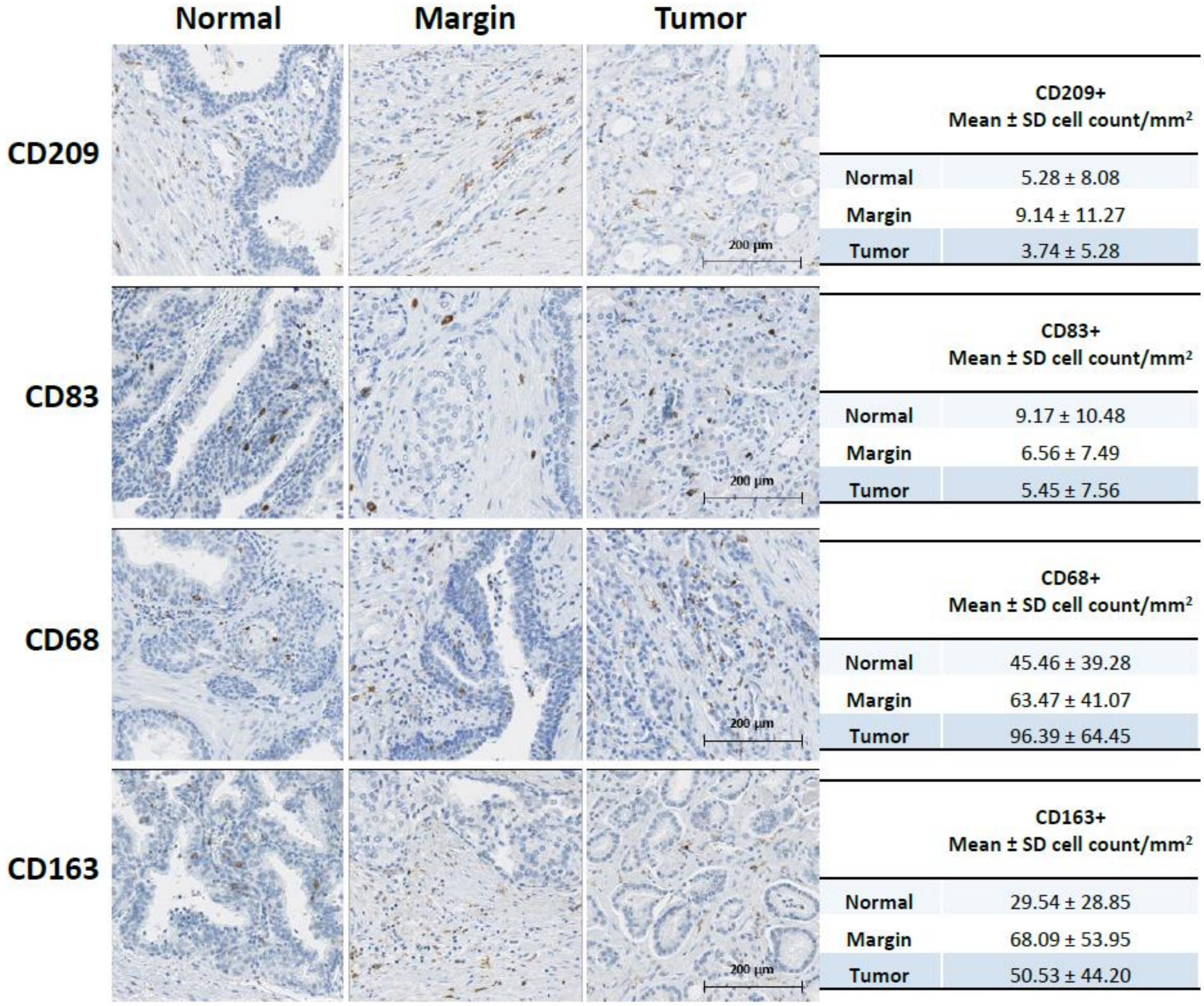

	CD209+ Mean ± SD cell count/mm²
Normal	5.28 ± 8.08
Margin	9.14 ± 11.27
Tumor	3.74 ± 5.28

	CD83+ Mean ± SD cell count/mm²
Normal	9.17 ± 10.48
Margin	6.56 ± 7.49
Tumor	5.45 ± 7.56

	CD68+ Mean ± SD cell count/mm²
Normal	45.46 ± 39.28
Margin	63.47 ± 41.07
Tumor	96.39 ± 64.45

	CD163+ Mean ± SD cell count/mm²
Normal	29.54 ± 28.85
Margin	68.09 ± 53.95
Tumor	50.53 ± 44.20

Supplementary Figure S1. Examples of staining for $CD209^+$ (iDC), $CD83^+$ (mDC), $CD68^+$ (total MΦ), and $CD163^+$ (M2 MΦ) cells in tumor, tumor margin, and normal-like adjacent epithelium areas as determined by immunohistochemistry. Magnification 20X. Scale bars = 200 µm. The mean number of cells/mm^2 ± SD in each compartment is also provided.
